# Elevated *p*CO_2_ Level Affects the Extracellular Polymer Metabolism of *Phaeodactylum tricornutum*

**DOI:** 10.3389/fmicb.2020.00339

**Published:** 2020-03-04

**Authors:** Wei Zhang, Xuexi Tang, Yingying Yang, Xin Zhang, Xinxin Zhang

**Affiliations:** ^1^Department of Marine Ecology, College of Marine Life Sciences, Ocean University of China, Qingdao, China; ^2^Laboratory for Marine Ecology and Environmental Science, Qingdao National Laboratory of Oceanology for Marine Science and Technology, Qingdao, China

**Keywords:** extracellular polymeric substances, *Phaeodactylum tricornutum*, elevated *p*CO_2_, adaptation, metabolic plasticity, RNA-seq

## Abstract

Extracellular polymeric substances (EPS) play an important role in diatom physiology and carbon biogeochemical cycling in marine ecosystems. Both the composition and yield of EPS in diatom cells can vary with environmental changes. However, information on intracellular pathways and controls of both biochemical and genetic of EPS is limited. Further, how such changes would affect their critical ecological roles in marine systems is also unclear. Here, we evaluated the physiological characteristics, EPS yields, EPS compositions, and gene expression levels of *Phaeodactylum tricornutum* under elevated *p*CO_2_ levels. Genes and pathways related to EPS metabolism in *P. tricornutum* were identified. Carbohydrate yields in different EPS fractions increased with elevated *p*CO_2_ exposure. Although the proportions of monosaccharide sugars among total sugars did not change, higher abundances of uronic acid were observed under high *p*CO_2_ conditions, suggesting the alterations of EPS composition. Elevated *p*CO_2_ increased PSII light energy conversion efficiency and carbon sequestration efficiency. The up-regulation of most genes involved in carbon fixation pathways led to increased growth and EPS release. RNA-Seq analysis revealed a number of genes and divergent alleles related to EPS production that were up-regulated by elevated *p*CO_2_ levels. Nucleotide diphosphate (NDP)-sugar activation and accelerated glycosylation could be responsible for more EPS responding to environmental signals. Further, NDP-sugar transporters exhibited increased expression levels, suggesting roles in EPS over-production. Overall, these results provide critical data for understanding the mechanisms of EPS production in diatoms and evaluating the metabolic plasticity of these organisms in response to environmental changes.

## Introduction

Extracellular polymeric substances (EPS) produced by microorganisms primarily comprise acid mucopolysaccharides (Mcconville et al., 1985), are ubiquitous in aquatic environments (Sutherland, 1999; Donot et al., 2012), and play important roles in cellular adhesion, signaling, and stress responses (Underwood and Paterson, 2003; Taylor et al., 2013). Diatoms are one of the most abundant photosynthetic organisms in oceans where they occupy diverse habitats and are one of the primary contributors of EPS pools in marine ecosystems. Polysaccharide-rich EPS released by diatoms can be utilized as a carbon source by bacteria, meiofauna, and macrofauna (Middelburg et al., 2000). Indeed, diatom EPS is an important component of marine dissolved organic matter (DOM) (Deming, 2010; Underwood et al., 2013) and is thus an integral component of the oceanic carbon cycle in addition to other cycles (Simon et al., 2002). Moreover, the formation of micro-environments by diatom EPS promotes microbial interactions within and around EPS matrices (Sonnenschein et al., 2011).

Extracellular polymeric substances are considered important adaptations of diatoms to their environments. For example, they can play an important role in the stability of intertidal flats and the formation of sediments (de Brouwer et al., 2005, 2002). Further, EPS form buffer zones between cells and environments, thereby helping diatom cells withstand low temperatures, desiccation, salinity stress, and toxicant exposure (Aslam et al., 2012; Steele et al., 2014). Concomitantly, EPS has the potential to interact with various ions or compounds via an abundance of polar functional groups, and can thereby alter the bioavailability and toxicity of compounds (Czaczyk and Myszka, 2007). The composition and structure of EPS varies in response to the growth environments of diatoms (Abdullahi et al., 2006; Ai et al., 2015). For example, algae respond to inorganic nutrient constraints, salinity, and low temperature stress by altering their EPS production rates as well as their chemical properties (Underwood et al., 2004; Abdullahi et al., 2006; Mishra and Jha, 2009). Moreover, studies have shown that the monosaccharide components, protein contents, molecular weights, and surface properties of EPS may affect their activities and counteract external interference (Calazans et al., 2000; Song et al., 2015).

During EPS synthesis, monosaccharides are converted to NDP-sugars by multiple pathways and then assembled into polysaccharides. This mechanism of production is evolutionarily conserved among prokaryotes and eukaryotes. The polysaccharides are further incorporated into EPS in the Golgi apparatus and then transported via vesicles to cellular membranes and excreted (Gügi et al., 2015; Aslam et al., 2018). Complex genetic networks are necessary to enable cells to flexibly respond to their environments by altering EPS characteristics (Underwood et al., 2004; Aslam et al., 2018). A recent study identified the metabolic pathways for EPS production in the polar diatom *Fragilariopsis cylindrus* that changed in response to temperature and salinity gradients, suggesting that their metabolic plasticity was attributable to molecular complexity (Aslam et al., 2018). However, it is unclear if these responses are characteristic of only psychrophilic algae like *F. cylindrus* or are instead common features of diatom taxa in other environments.

Increasing CO_2_ concentrations alters the chemical nature of seawater carbonates as well as the availability and toxicity of nutrients (Millero et al., 2009). Marine organisms such as phytoplankton, mussels, and fish are particularly sensitive to changes in carbonate chemistry, and elevated *p*CO_2_ levels could have profound effects on marine ecosystems (Doney et al., 2009). Thus, the possible biological consequences of elevated *p*CO_2_ levels has recently received extensive research attention. In particular, several studies have reported the effects of elevated *p*CO_2_ levels on diatoms at the physiological and ecological scales (Wu et al., 2010; Gao and Campbell, 2014; Shi et al., 2019). However, few studies have evaluated the molecular mechanisms underlying diatom responses to elevated *p*CO_2_ levels. Indeed, many mechanisms by which diatoms adapt and respond to elevated *p*CO_2_ levels remain unknown.

Despite that EPS plays important roles in the responses of diatoms to environmental changes, variation in diatom EPS in response to elevated *p*CO_2_ levels is unknown. Further, the commonalities of EPS synthesis and mechanisms of metabolism have not been clarified among different diatom taxa from different environments. Given the above lack of information, the response of EPS characteristics to elevated *p*CO_2_ levels in addition to the EPS metabolic pathways of *Phaeodactylum tricornutum* were investigated in the present study. *P. tricornutum* is a model species for diatom biology with clear genetic background, unique evolutionary status and properties that can be routinely transformed (Scala et al., 2002; Kooistra et al., 2003). Further, the genetic background of *P. tricornutum* has been described in detail and was thus leveraged to investigate the effects of elevated *p*CO_2_ levels on the organism. *P. tricornutum* was cultured under different *p*CO_2_ levels for approximately 200 generations, and several parameters were investigated among treatments including changes in growth characteristics, morphology, photophysiology, and the differences in carbohydrate yields within EPS. Metabolic pathways for EPS production were also evaluated in context of the algal response to elevated *p*CO_2_ levels. The results of this study provide important baseline data to develop an understanding of the regulation of key genes involved in the metabolic plasticity of diatoms in response to environmental signals.

## Materials and Methods

### Algal Cultures and the Elevated *p*CO_2_ Experimental System

The *P. tricornutum* (Bacillariophyta, Pennatae, NPECC640) culture was obtained from the Algal Center of the Institute of Oceanology of the Chinese Academy of Sciences. The cells were cultured in modified f/2 medium (Guillard, 1975) at 20 ± 1°C, with illumination at 120 μmol photon m^–2^ s^–1^ under 12 h light:12 h dark cycles. Flasks were shaken twice a day at fixed times. Cells in the mid-logarithmic growth phase were used for assays. All experiments were conducted in triplicate in 500 mL sterilized and acid-washed Erlenmeyer flasks containing 350 mL of medium. The equipment used in this study was similar to that used in previous investigations (Hutchins et al., 2007; Wu et al., 2010; Fu et al., 2012; Jin et al., 2015). Prior to inoculation, the media were infused with different *p*CO_2_ concentrations. The low CO_2_ treatment media was bubbled with ambient air (400 ppmv, LC, pH_NBS_ 8.16), while the high CO_2_ treatment media was bubbled with pre-mixed air-CO_2_ mixtures (1,000 ppmv, HC, pH_NBS_ 7.82) using a plant growth CO_2_ chamber (HP400G-D, Ruihua Instrument & Equipment, Ltd., Wuhan, China), controlling the CO_2_ concentration with a less than 3% variation. Triplicates of LC and HC cultures were all started with the same initial cell densities of 1 × 10^4^ cells mL^–1^. Every 24 h, a portion of the culture was removed and the remaining culture has refreshed with *p*CO_2_ and a pH adjusting medium to maintain the desired pH and *p*CO_2_ values within a range of less than 0.5%. Semi-continuous cultivation was used to maintain pH stability over the *P. tricornutum* growth period (Jin et al., 2015). The cultures were grown semi-continuously and were pre-acclimated to experimental conditions for more than 30 generations. Cultures were harvested after 200 generations of semi-continuous incubation. Significant differences were observed in the carbonate systems among different cultures (Supplementary Table S1).

### Cell Growth Measurements and Morphological Observations

Cell densities were measured using a particle count and size analyzer (Z2 Coulter, Beckman). The specific growth rate was calculated as follows: μ = (ln *N*_1_–ln *N*_0_) × (*t*_1_–*t*_0_)^–1^, where μ (d^–1^) is the specific growth rate, and *N*_0_ and *N*_1_ are the cell numbers at times *t*_0_ and *t*_1_, respectively. After filtering 10 mL of the culture, algal cells were collected and dehydrated, followed by spraying with gold, and placing on a scanning microscope to observe cell morphology. The scanning electron microscope (SEM; model XL-30, Phillips, Eindhoven, Netherlands) was operated at 2.0 kV to observe EPS and cell characteristics.

### Determination of Photophysiology

Methanol was used as a solvent to extract pigments from *P. tricornutum* (Macías-Sánchez et al., 2007). Chlorophyll-*a* (Chl *a*) concentrations were determined spectrophotometrically as follows: Chl *a* = 16.29 × (A665–A750) – 8.54 × (A652–A750), where A652, A665, and A750 represent the absorbances of the methanol extracts at 665, 652, and 750 nm, respectively (Porra et al., 1989). As we did not use the Jeffrey and Humphrey equation (1975) which also corrects for chlorophyll-*c* present in diatoms, these values can only be taken as a proxy for the amount of chlorophyll-*a.* The maximum quantum yields (*F*_v_/*F*_m_) were measured with a Xenon-Pulse Amplitude Modulated Fluorometer (Water-PAM fluorometer, Walz, Effeltrich, Germany) at the middle of the light period of growth. Before the determination, algal cells were dark acclimated for 15 min to obtain completely oxidative PSII reaction centers. Measurement of photosynthetically driven carbon fixation in addition to dark respiration were conducted as described previously (Wu et al., 2010; Hong et al., 2017).

### Extraction of Carbohydrate Fractions and Compositional Analysis

Based on previously established sequential extraction methods (Abdullahi et al., 2006), 50 mL of algal culture was used to fractionate the diatom EPS into: (I) a soluble (colloidal) fraction containing both colloidal carbohydrate extractions (CL) a polymeric fraction (cEPS fraction); (II) a hot water (HW)-extracted carbohydrate fraction mainly consisting of intracellular storage polysaccharides; (III) a hot bicarbonate (HB)-extracted fraction (relative to gelatinous and water-insoluble extracellular polysaccharides) and (IV) a hot alkali extraction (HA) liberating EPS associated with the silica frustules. The concentrations of carbohydrates in each fraction were determined using a phenol sulfuric acid assay, a uronic acid assay with a standard carbazole assay, and neutral monosaccharide compositions were determined by gas chromatography coupled to mass spectrometry. Monosaccharide compositions within carbohydrate fractions were compared using ANOSIM and SIMPER statistical tests (Primer v.6, Plymouth, United Kingdom).

### RNA Extraction, Library Preparation, and Illumina Sequencing

*Phaeodactylum tricornutum* cells in the middle of the light period from three biological replicates were harvested after 200 generations of semi-continuous incubation using vacuum filtration on 1.2 μm polycarbonate filters (Millipore, United States), quickly frozen in liquid nitrogen and maintained at −80°C until use. Total RNA was extracted from frozen cell pellets using the TRIzol^®^ Reagent (Invitrogen, Carlsbad, CA, United States) according to the manufacturer’s instructions, and DNase (Sigma-Aldrich, St. Louis, MO, United States) was added to digest the DNA. RNA integrity was evaluated using an Agilent 2100 Bioanalyzer (Agilent Technologies, Santa Clara, CA, United States). Extracts with RNA Integrity Number (RIN) ≥ 7 were used for subsequent sequencing library preparation (Supplementary Figure S2). The libraries were constructed using the TruSeq Stranded mRNA LTSample Prep Kit (Illumina, San Diego, CA, United States) according to the manufacturer’s instructions. The libraries were then sequenced on the Illumina sequencing platform (HiSeqTM 2500) by generating 150 bp paired-end reads.

### RNA-seq Data Assembly and Analysis

Transcriptomic sequencing and analyses were conducted by OE Biotech, Co., Ltd. (Shanghai, China). Raw data were quality filtered using Trimmomatic v.0.36 (Bolger et al., 2014). Specifically, reads containing poly-N bases and low-quality reads (quality values < 19 accounting for more than 15.0% of the total bases) were removed to obtain clean reads. The reference genome of *P. tricornutum* was downloaded and used for read-mapping^1^. Clean reads were mapped to the reference genome using hisat2 (Kim et al., 2015). The Fragments Per Kilobase Million (FPKM) value for each gene was calculated using cufflinks (Trapnell et al., 2010), and the read counts of each gene were obtained by htseq-count (Anders et al., 2015). Differentially expressed genes (DEGs) were then identified using the DESeq (Anders and Huber, 2012) R package functions ‘estimate size factors’ and ‘nbinomTest.’ Genes with fold change ratio (HC/LC) ≥ 2 (*P*_adj_ ≤ 0.05) and fold change ratio ≤ 0.5 (*P*_adj_ ≤ 0.05) were defined as “up-regulated genes” and “down-regulated genes,” respectively. Hierarchical cluster analysis of DEGs was used to explore patterns of gene expression. Principal component analysis (PCA) and heat map of correlation analysis were then performed based on differences in gene expression levels to investigate overall differences in samples, evaluate inter-sample relationships, and validate experimental designs.

### Identification and Hierarchical Clustering Analysis of Carbohydrate Metabolism-Related Proteins

Carbohydrate active enzymes (CAZys) were identified and annotated based on comparisons to the CAZy database^2^ and then clustered with hierarchical clustering using their FPKM values. A one minus Pearson’s correlation distance metric and the average linking method was applied to cluster genes and results visualized using the Bioconductor R ComplexHeatmap package (Gu et al., 2016).

### Reconstruction of EPS Production Pathways

Extracellular polymeric substances contains abundant polysaccharides and glycans that are assembled and modified within the inner membrane system. The genes involved in the synthesis and metabolism of sugars as well as those involved in EPS assembly and Golgi modification were retrieved from the *P. tricornutum* genome based on comparison against annotations in the GO, KEGG, KOG, and CAZy databases. In addition, hidden Markov models and Hmmsearch-3.2.1 (Mitchell et al., 2018) were used to identify nucleotide glycosyltransferases with the nucleotide-sugar transporter (PF04142) model in the *P. tricornutum* genome. Nucleotide glycosyltransferases transport NDP- sugars from the cytoplasm to the Golgi apparatus. Thus, all metabolic genes that were identified were surveyed for the presence of signal peptides using SignalP (Nielsen, 2017). Those proteins that were predicted to be cytosolic, endoplasmic reticulum (ER) associated, or Golgi enzymes that also lacked any conserved plastids (ASAF) or mitochondrial target sequences, were selected for further analysis.

### Reverse Transcription Quantitative Real-Time PCR

Reverse transcription quantitative PCR (RT-qPCR) was used to validate the transcriptomic data using primers listed in Supplementary Table S3. Polyadenylated RNA was converted to cDNA with the PrimeScriptTM II Reverse Transcriptase (TaKaRa, Tokyo, Japan). The RT products were then used as template for qPCR. qPCR reactions were performed on an FTC2000 instrument (Funglyn Biotech, Inc., Toronto, ON, Canada) using SYBR Green I mix (TaKaRa, Tokyo, Japan) in 96-well plates according to the manufacturer’s recommendations. The TATA box binding protein and ribosomal protein small subunit 30S (RPS) housekeeping genes (Siaut et al., 2007) were used as references to calibrate expression data. Three biological replicates were conducted for each qPCR reaction.

### Data Analysis

All data are expressed as the mean ± SE (*n* = 3). Statistical differences were analyzed by one-way ANOVA tests performed with the SPSS software program (version 22.0) followed by a Tukey’s multiple comparisons adjustment, with statistical significance set at *P* < 0.05. All data were tested for normality with Kolmogorov–Smirnov tests and homogeneity of variance with Levene’s tests. Redundancy analysis (RDA) was performed using the ‘vegan’ R package (Oksanen et al., 2003) to identify statistically significant relationships between physiological, biochemical, and transcriptomic datasets. The RDA results were visualized using the ggplot2 R package (Wickham, 2016). Detailed analytical protocols are provided in the Supplementary Information. The RNA-seq data were deposited in the ArrayExpress database^3^ under accession number E-MTAB-8351. Supplementary materials can be found at https://issues.pangaea.de/browse/PDI-22044.

## Results

### *P. tricornutum* Growth, Morphology, and Photophysiology Under Different *p*CO_2_ Treatments

**FIGURE 1:**
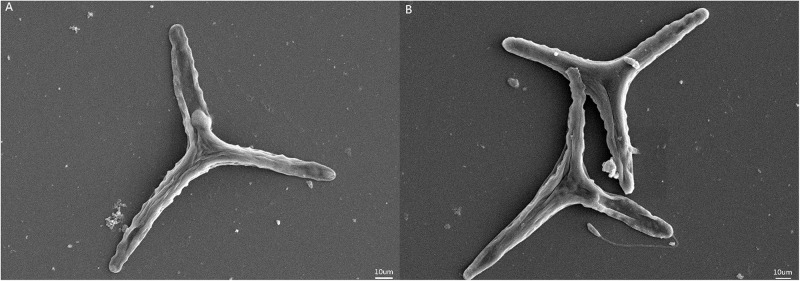
SEM images of *Phaeodactylum tricornutum* cells under different *p*CO_2_ conditions. **(A)** SEM of *P. tricornutum* in the LC treatment. **(B)** SEM of *P. tricornutum* in the HC treatment.

Ann increase in the specific growth rate (2.4%) was observed for populations in the HC condition (Table 1). *F_*v*_/F_*m*_* values were higher for the HC condition cells, but significant differences were not observed in pigment contents of cells in the HC and LC treatments. The photosynthetically driven carbon fixation rate and the respiration rate significantly increased (ANOVA, *P* < 0.05) by 13.5% and 33.6% in the HC treatment, respectively. SEM indicated that the preferential morphotype of *P. tricornutum* in both treatments was triradiate (Figure 1), which accounted for more than 70.0% of the cellular morphotypes. Morphotype differences were not observed between the LC and HC conditions, but more loosely adhered mucilage occurred near the cells in the HC treatment.

**TABLE 1:** The physiological characteristics of *P. tricornutum* after 200 generations of semi-continuous incubation under LC and HC conditions.

**Treatment**	**μ(d^–1^)**	**Chl *a* (pg cell^–1^)**	***F_*v*_/F_*m*_***	**Photosynthetic carbon fixation [μmol C (μg chl *a*)^–1^ h^–1^]**	**Dark respiration [μmol O_2_(μg chl *a*) ^–1^ h^–1^]**
LC	1.22 ± 0.01^a^	0.22 ± 0.01	0.59 ± 0.01^a^	0.37 ± 0.02^a^	0.098 ± 0.007^a^
HC	1.25 ± 0.01^b^	0.24 ± 0.02	0.62 ± 0.01^b^	0.42 ± 0.01^b^	0.131 ± 0.010^b^

*μ, specific growth, Chl *a*, Chlorophyll-*a*, and *F_*v*_/F_*m*_*, maximum quantum yield. The values are means ± SE, and different superscript letters indicate statistically significant differences between LC and HC conditions.*

### Variation in the Yield and Chemical Composition of Carbohydrates

**FIGURE 2:**
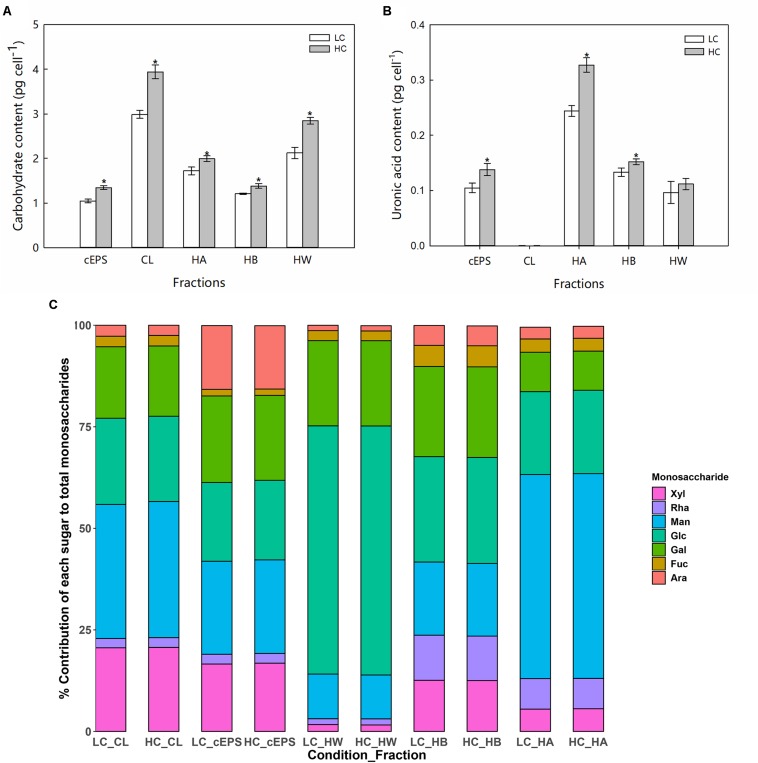
Carbohydrate content (**A**, pg cell^–1^), uronic acid content (**B**, pg cell^–1^), and **(C)** monosaccharide compositions of different EPS fractions from cells grown in the LC and HC treatments. “*” represents significant difference between groups (ANOVA, *P* < 0.05). Data represent averages of three replicates, with the standard errors shown. CL, colloidal; cEPS, colloidal extracellular polymeric substances; HW, hot water soluble; HB, hot bicarbonate soluble; HA, hot alkali soluble; Glc, Glucose; Ara, Arabinose; Xyl, Xylose; Fuc, Fucose; Man, Mannose; Gal, galactose; Rha, Rhamnose.

Compared with the LC treatment, significant increases in carbohydrate yields (ANOVA, *P* < 0.05) were observed for each carbohydrate fraction of *P. tricornutum* in the HC treatment (Figure 2A). The CL carbohydrate fraction increased by 31.8% in the HC condition, while the cEPS, HW, HB, and HA fractions increased by 28.4, 34.2, 14.1, and 15.9%, respectively. In particular, the HC treatment conditions stimulated the production of uronic acid in *P. tricornutum.* Compared against the LC conditions, the uronic acid content of the CL, HW, and HB fractions increased significantly in the HC conditions by 1. 31-, 1. 13-, and 1.41-fold, respectively (Figure 2B). Monosaccharide analysis indicated that mannose was the most abundant monosaccharide component of the CL, cEPS, and HA fractions. In contrast, HW fractions were enriched in glucose, with glucose generally exceeding 50.0% of the total monosaccharides of those fractions. Galactose was the second most abundant monosaccharide after glucose within the HB carbohydrate fractions. The relative abundances of fucose, arabinose, and rhamnose were lower than those of other monosaccharides. The concentrations of each monosaccharide increased in the HC treatments, while the proportion of monosaccharides of the total sugar content did not significantly change between treatments (Figure 2C).

### The *P. tricornutum* Transcriptomic Response to High *p*CO_2_ Conditions

**FIGURE 3:**
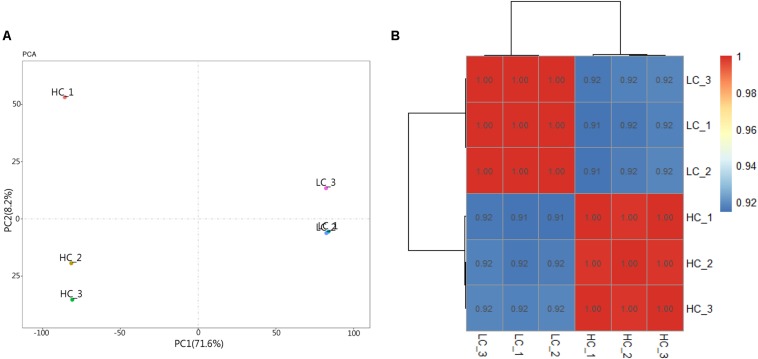
Principal component analysis (PCA) and heat map of correlation analysis results of samples. **(A)** Principal component analysis (PCA) result. **(B)** Heat map of correlation analysis results of samples. The legend represents the value of the correlation coefficient, and the color changing from blue to red corresponds to the correlation coefficient from small to large. Triplicate samples are shown for the high (HC) and low (LC) *p*CO_2_ conditions.

**FIGURE 4:**
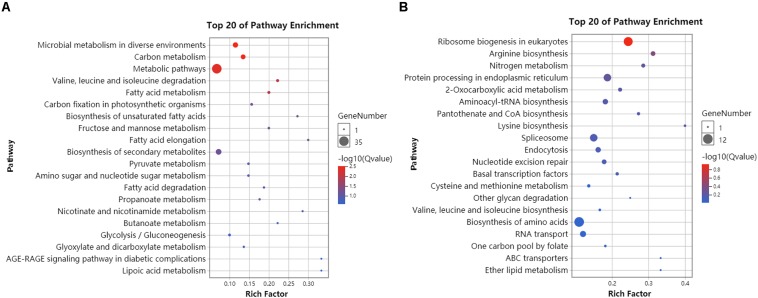
KEGG enrichment analysis of differentially expressed genes in HC compared to LC conditions. **(A)** The 20 most abundant KEGG pathways that were associated with significantly up-regulated genes (HC/LC). **(B)** The 20 most abundant KEGG pathways that were associated with significantly down-regulated genes (HC/LC). Circle size corresponds to greater numbers of enriched genes within that pathway. Circle colors indicate the enrichment scores for that pathway. Compared with LC condition, genes involved in carbon and fatty acid metabolism are up-regulated, while those involving protein synthesis are down-regulated in HC condition.

RNA-seq data generated from the HC and LC treatment cells were assembled and mapped to the *P. tricornutum* genome. The sequencing analysis of six samples yielded a total of 38.59 Gbp of clean data, with an average of 6.43 Gbp of data per sample. Q30 bases comprised 93.6% of the clean read data and gene coverage was 98.9% or higher. The average total mapping rate of RNA-seq data was about 90.1% (Supplementary Table S2). Thus, the data was of high quality and appropriate for evaluating gene expression in response to HC conditions. Principal component analysis (PCA) of differences in gene expression levels revealed a clear distinction between the two conditions (Figure 3A). The difference between the HC group and the LC group is mainly in the PC1 dimension, which indicates that the difference between the two groups is huge and greatly exceeds the difference within the group. The correlation coefficients among the samples within groups were 1 (Figure 3B), suggested a good correlation. A total of 2,106 protein coding genes were differentially expressed in the HC condition with 990 up-regulated genes and 1,116 down-regulated genes compared to cells in the LC condition. The up-regulated genes were significantly associated with carbon metabolism (ko01200), carbon fixation in photosynthetic organisms (ko00710), fructose and mannose metabolism (ko00051), Glycolysis/Gluconeogenesis(ko00010) and amino sugar and NDP-sugar metabolism (ko00520), among other pathways (Figure 4A). Down-regulated genes were significantly associated with ribosome biogenesis in eukaryotes (ko03008), RNA transport (ko03013), and spliceosome (ko03040), among other pathways (Figure 4B). Compared with LC condition, genes involved in carbon and fatty acid metabolism are up-regulated, while those involving protein synthesis are down-regulated in HC condition. A high level of concordance was observed between the transcriptomic and RT-qPCR data, thus validating the robustness of the sequencing data (Supplementary Table S3).

### Identification of Genes Involved in the Production of EPS

A total of 269 genes encoding carbohydrate active enzymes (CAZy) were identified in the genome including 53 glycoside hydrolases (GHs), 161 glycosyltransferases (GTs), 36 carbohydrate esterases (GEs), and 20 carbohydrate binding modules (CBMs). Four genes (Phatr3_J35856, Phatr3_J48916, Phatr3_J50429, Phatr3_Jdraft1777) encoding carbohydrate active enzymes were not detected in the expression for both treatment conditions. Cluster analysis of the CAZy protein expression data are shown in Supplementary Figure S1, after removal of genes not detected in both treatment conditions. The CAZy proteins could be divided into three clusters based on expression patterns, with 64 down-regulated genes in the first cluster, eight genes with expressions that did not vary between treatments in the second cluster, and the remaining genes belonging to the third cluster. The HC treatment resulted in up-regulation of 72.8% of the genes encoding CAZys. In addition, eight genes encoding nucleotide-sugar transporters were identified by Hmmsearch, with seven up-regulated. Phatr3_EG01605 was the most significantly up-regulated gene and encoded a NDP-sugar transporter.

**FIGURE 5:**
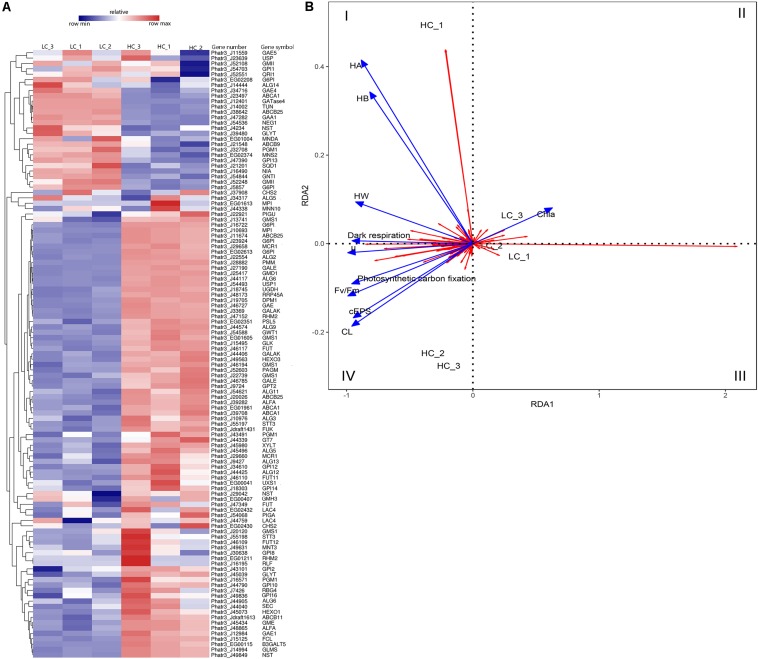
Differences in the expression of EPS metabolism-related genes among LC and HC grown cells. **(A)** Heatmap analysis of EPS metabolism-related gene expression. Hierarchical cluster analysis of expression values [average fragments per million transcripts/million map reads (FPKM)] was conducted for 115 EPS metabolism-related genes. The gene identifiers, abbreviations, and annotations are shown in Supplementary Table S4. The expression levels of genes correspond to the relative expression scale above the heatmap. The dendrogram was generated using full-linkage clustering based on the Pearson correlation distance matrix of gene expression level differences. **(B)** Redundancy analysis (RDA) showing association among growth characteristics and gene expression differences. The blue colored arrows represent different physiological indicators, and the red arrows represent different genes. Arrows are given in the direction of their highest correlation to the sample ordination space, with angle lengths indicating greater correlations in that direction. μ, specific growth; Chl *a*, Chlorophyll-*a*; *F_*v*_/F_*m*_*, maximum quantum yield; CL, colloidal fraction carbohydrate content; cEPS, colloidal extracellular polymeric substances fraction carbohydrate content; HW, hot water-soluble fraction carbohydrate content; HB, hot bicarbonate soluble fraction carbohydrate content; HA, hot alkali soluble fraction carbohydrate content.

**FIGURE 6:**
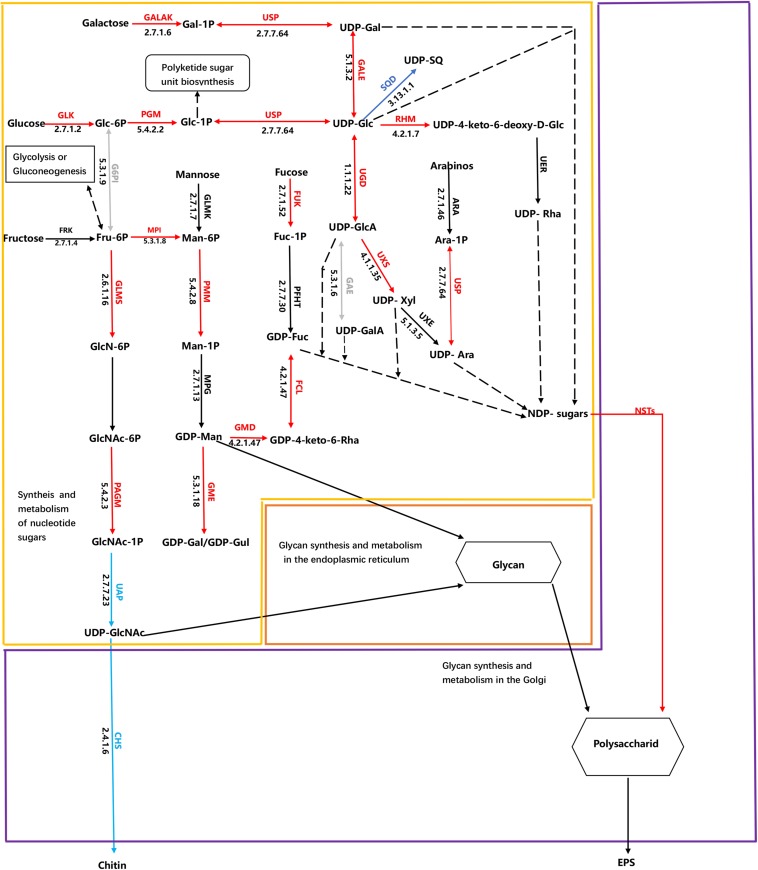
Schematic of metabolic pathways associated with EPS production in *Phaeodactylum tricornutum.* The arrows are colored according to changes in HC conditions in metabolic processes compared to LC conditions. The red arrows indicate up-regulation of genes involved in EPS synthesis, while blue arrows indicate down-regulated genes in EPS biosynthesis. Green arrows indicate that differences in expression were not significant between treatments, while gray arrows indicate that gene expression differences were significant, but some are up-regulated. The black arrows indicate that the pathway has not been identified, while black dashed lines indicate that the UDP and GDP sugars are both NDP-sugars. Expressional patterns for identified enzymes and isoenzymes are shown together with ENSEMBL protein identifiers. Chemical compound abbreviations are as follows: Glc-6P, glucose-6-phosphate; Glc-1P, glucose-1-phosphate; Fru-6P, fructose-6-phosphate; FUC-1P, Fucose 1-phosphate; Ara-1p, Arabinose 1-phosphate; UDP-Glc, UDP-glucose; Gal-1P, galactose-1-phosphate; UDP-Gal, UDP-galactose; Man-6P, mannose-6-phosphate; Man-1P, mannose-1-phosphate; GDP-Man, GDP-mannose; GDP-Fuc, GDP-fructose; GlcN-6P, glucosamine-6-phosphate; GlcNAc-6P, *N*-acetylglucosamine-6-phosphate; GlcNAc-1P, *N*-acetylglucosamine-1-phosphate; UDP-GlcNAc, UDP-*N*-acetylglucosamine; UDP-GlcA, UDP-glucuronic acid (glucuronate); UDP-SQ, UDP-6-sulfoquinovose; UDP-Xyl, UDP-xylose; UDP-Ara, UDP –arabinose; UDP-Rha, UDP-rhamnose; GDP-Gul, GDP –gulose; GDP-Gal, GDP- galactose; GDP-4-keto-6-deoxy-D-man, GDP-4-keto-6-deoxy-D-mannose; UDP-4-keto-6-deoxy-D-Glc, UDP-4-keto-6-deoxy-D-glucose. Enzyme abbreviations are also as follows: GLK, glucokinase; PGM, phosphoglucomutase; GALAK, galactokinase; USP, UDP-sugar pyrophosphorylase; GALE, UDP-glucose 4-epimerase; RHM, UDP-glucose 4,6-dehydratase G6PI glucose-6-phosphate isomerase; MPI, mannose-6-phosphate isomerase; UGD, UDP-glucose-6-dehydrogenase; SQD, UDP-sulfoquinovose synthase; GLMS, glutamine-fructose-6-phosphatetransaminase; FRK, fructokinase; UAP, UDP-*N*-acetylglucosamine diphosphorylase; PAGM, phosphoacetylglucosaminemutase; CHS, chitin synthase; PMM, phosphomannomutase; FUK, fucokinase; GMD, GDP-mannose 4,6-dehydratase; GME, GDP-D-mannose 3′ 5′-epimerase; FCL, GDP-L-fucose synthase; UXS, UDP-glucuronate decarboxylase; NST, Nucleotide-sugar transporter; GMS, UDP-galactose transmembrane transporter; ABC, ABC transporter.

Cluster analysis was conducted using the differences in expression data for 115 protein-coding genes related to EPS metabolism, including divergent allelic gene copies involved in synthesis pathways of NDP-sugars, NDP-sugar transporters, and glycan synthesis (Figure 5A). Many of the proteins were encoded by more than one gene, including for example, glucose-6-phosphate isomerase and UDP-Glucose-Pyrophosphorylase. Most of the genes involved in NDP-sugars synthesis was up-regulated. Phatr3_EG02613, Phatr3_J10693, Phatr3_J47152, and Phatr3_J25417 were the most significantly up-regulated genes and encoded phosphoglucomutase, mannose-6-phosphate isomerase, UDP-glucose 4,6-dehydratase, and GDP-mannose 4,6-dehydratase, respectively. These enzymes are important for glucose and mannose activation into NDP-sugars (Figure 6). GDP-mannose can be directly used as a substrate in the endoplasmic reticulum and undergoes modification by several mannosyltransferase (beta-1,4-mannosyltransferase, alpha-1,3/alpha-1,6-mannosyltransferase, alpha-1,3-mannosyltransferase, alpha-1,2-mannosyltransferase, alpha-1,2-mannosyltransferase, etc.), before being assembled into polysaccharides. Other NDP-sugars are transported by NDP-sugar transferases to the Golgi apparatus as substrates, assembled, and then modified into polysaccharides or glycans. In addition, the polysaccharides in the endoplasmic reticulum also enter the Golgi apparatus for further assembly and processing. Most of the genes involved in the assembly and modification of EPS in the Golgi apparatus were up-regulated, although Phatr3_J34317 and Phatr3_J54844 were significantly down-regulated. These genes encode dolichol phosphate glucosyltransferase and alpha-1,3-mannosyl-glycoprotein-2-beta-*N*-acetylglucosaminyltransferase, respectively.

### Relationship Between Gene Expression and the Physiological Status of Diatoms

Positive correlations were observed between diatom growth rate, *F_*v*_/F_*m*_* values, the photosynthetic carbon fixation rate, and the dark respiration rate, and they were all positively correlated with EPS yield (Figure 5B). The expression levels of Phatr3_J47152 (encoding UDP-glucose 4,6-dehydratase), Phatr3_J25417 (encoding GDP-mannose 4,6-dehydratase), and Phatr3_J27190 (encoding UDP-glucose 4-epimerase) were significantly and positively correlated with diatom growth rate, *F_*v*_/F_*m*_*, the photosynthetic carbon fixation rate, the dark respiration rate, CL products, and cEPS products. RDA results showed that genes within quadrant one (I) were positively correlated with carbohydrate production of HA, HB, HW, and also positively correlated with dark respiration. The genes in quadrants two and three (II and III) were positively correlated with Chl *a* content. Lastly, the genes in quadrant four (IV) were positively correlated with diatom growth rate, *F_*v*_/F_*m*_*, photosynthetic carbon fixation rate, and dark respiration rate, in addition to being positively correlated with carbohydrate yields of cEPS and CL.

## Discussion

### Roles of EPS Released by Diatoms in Response to Environmental Changes

Diatoms produce diverse EPS compounds that vary in structure and sugar composition depending on environmental conditions. The present study reports the first investigation of elevated *p*CO_2_ conditions on EPS released by diatoms. The carbohydrate yields of several EPS fractions (CL, cEPS, HA, and HB) were significantly higher under high CO_2_ (HC) conditions. Monosaccharide compositions influence the functional properties of exopolysaccharides, and ultimately determine the rheological properties of EPS, like gel formation (Decho and Gutierrez, 2017). To evaluate the effects of HC conditions on EPS structures, the relative abundances of neutral sugar monosaccharides were determined in the CL-, cEPS-, HB-, and HA-polymers after HC treatment. Complex monosaccharide profiles were clearly observed in the aforementioned EPS fractions produced by *P. tricornutum* (Figure 2C), consistent with previous results (Abdullahi et al., 2006). Compared with the LC treatment cells, there was no significant difference was observed in the relative proportions of monosaccharides among the total sugar contents in the HC treatments. In addition, total uronic acid content, which are polymer substituents that also influence physical characteristics, differed considerably between the LC and HC treatments, with increased ionic (uronic) groups observed in algal cells in the HC treatment. Polysaccharides with high proportions of uronic acid often contain an abundance of negatively charged ions and exhibit more available binding sites, leading to greater intra- and inter-chain associations within the polymer or with neighboring polymer groups (Magaletti et al., 2004; Kaplan, 2013). Thus, these results preliminarily suggested that elevated *p*CO_2_ conditions significantly increased EPS production in *P. tricornutum* cells and altered their functional properties.

The secretion of highly complex EPS may function to produce protective cell coatings due to carbohydrate-rich polymers undergoing reversible phase transitions from soluble forms into hydrated forms, followed by condensation into gels in response to environmental changes in ion concentrations, pH, and temperatures (Decho and Luoma, 1994). For example, the sea-ice diatom *F. cylindrus* reduced growth, but increased yields of EPS under incrementally colder conditions, while an association between EPS, freezing, and cell survival was also observed (Aslam et al., 2012). These observations coincide with the significant increases in polymers observed in this study in response to HC conditions. In addition, microscopic observations (Figure 1) revealed thicker mucilage coatings around *P. tricornutum* cells in the HC treatments. The excess production of EPS has been previously suggested to be due to unbalanced metabolism, wherein carbon-rich polysaccharides are released from cells because of excessive photosynthetic production beyond what is necessary for growth and maintenance of homeostasis (Berman-Frank and Dubinsky, 1999; Hessen and Anderson, 2008). HC treatment resulted in increased photosynthetic carbon fixation rates of *P. tricornutum* with up-regulation of expression levels for most genes involved in carbon-fixation pathways. Moreover, the PSII light energy conversion efficiency (*F_*v*_/F_*m*_*) of *P. tricornutum* grown under HC conditions was also higher, which would serve to continuously provide reductant and ATP for downstream CO_2_ assimilation. Overall, these results suggested that more organic carbon was generated in *P. tricornutum* cells in the HC conditions, which could also further enhance their growth. An increase in the growth of algal cells under HC conditions was observed (2.4%), although increased photosynthetic carbon fixation in this treatment was more pronounced (13.5%). These contrasting results may indicate that the fixed carbon could also have been released as EPS to meet the demand for basic somatic growth.

Elevated environmental CO_2_ concentrations may facilitate carbon fixation by phytoplankton, as suggested by several studies of algal cells (Wu et al., 2010; Shi et al., 2019), including the present study. Thus, it is generally expected that increased seawater CO_2_ concentrations will enhance marine primary productivity. However, these results also suggest that a fraction of this fixed carbon could be released into seawater via EPS, thereby introduction further uncertainty to the carbon-based response of diatoms to increased ocean CO_2_ concentrations. EPS release rival that of other types of DOM that would be released in terms of carbon budgets is a question worthy exploring. In addition, the interactive effects of seawater pH and other environmental divers introduce further uncertainty in these predictions (Gao et al., 2012; Riebesell and Gattuso, 2014). Thus, the effects of EPS over-production on oceanic primary productivity and the biogeochemical cycling of carbon in marine ecosystems requires further investigation.

### Nucleotide Diphosphate (NDP)-Sugar Activation and Accelerated Glycosylation Might Be Responsible for HC-Induced EPS Overproduction in *P. tricornutum*

Differential patterns of gene expression alter cellular metabolism and ultimately result in EPS production in diatoms (Aslam et al., 2018). To evaluate the underlying mechanisms for the high-plasticity in carbohydrate and EPS physiology in *P. tricornutum*, genes and divergent alleles related to EPS production were identified using transcriptomic profiling. A total of 74.0% of these genes had up-regulated expression profiles under HC conditions. In addition, the expression patterns were correlated with physiological and biochemical properties of the cells under HC conditions (RDA analysis, Figure 5B).

Two potential responses of *P. tricornutum* EPS metabolism to elevated *p*CO_2_ were identified that could explain the over-release of EPS. First, the pathway of NDP-sugar synthesis and metabolism is a key step in EPS production and one of the substrates for nucleotide acid sugar synthesis is glucose, which is produced during photosynthesis (Figure 6, yellow frame). Most of the genes involved in the synthesis and metabolism of glucose and nucleotide sugars were up-regulated in the HC treatment cells. Glucose can be used to generate ATP (i.e., through glycolysis, oxidative phosphorylation, and the TCA cycle) and storage compounds like chrysolaminarin, and are also converted to other sugars and derivatives (Kroth et al., 2008). In particular, glucose is converted to UDP-glucose by glucokinase, glucose phosphate mutase (PGM), and UDP-*N-*acetylglucosamine diphosphorylase. UDP-glucose is a nucleotide sugar that can then be converted to UDP-galactose, and also converted to UDP-glucuronic acid, and further modified to UDP-xylose and UDP-arabinose. Importantly, UDP-glucose conversion is the only source of UDP-xylose and UDP-rhamnose, with no genes encoding arabinose kinase that could convert the monosaccharide to UDP-xylose and UDP-rhamnose being identified in the *P. tricornutum* genome. Nevertheless, rhamnose and xylose content is suboptimal for EPS production compared to glucose (Figure 2C). UDP-glucose 6-dehydrogenase promotes the conversion of UDP-glucose to UDP-glucuronic acid and its expression levels were elevated in the HC treatment cells, which could explain the increased uronic acid content in the EPS of those cells. Glucose-1-phosphate can enter the polyketide sugar unit biosynthesis pathway (ko00523) as a substrate, where it is converted to a dTDP-sugar and enters other metabolic pathways. A gene encoding fructokinase that converts fructose to fructose-6-phosphate was not observed in *P. tricornutum*, although fructokinase orthologs have been reported in the diatoms *Thalassiosira* and *Fragilariopsis*. Thus, this reaction may be catalyzed by an unidentified enzyme. An alternative possibility is that fructose-6-phosphate may derive from glycolysis or gluconeogenesis in *P. tricornutum* since genes in the glycolysis/gluconeogenesis pathways (ko00010) that convert 3-phosphoglycerate to fructose-6-phosphate, were significantly up-regulated in cells within the HC treatment. Fructose-6-phosphate can be further converted to glucosamine-6-phosphate, mannose-6-phosphate, or glucose-6-phosphate. Indeed, the expression levels of genes encoding glutamine-fructose-6-phosphate transaminase, mannose-6-phosphate isomerase, and glucose-6-phosphate isomerase, which are all responsible for these reactions were up-regulated in cells of the HC treatment. Glucosamine-6-phosphate is converted to chitosan by phosphor-acetylglucosamine mutase, UDP-*N*-acetylglucosamine, and chitin synthase. However, the expression levels of the encoded chitin synthase gene and the gene encoding UDP-*N-*acetylglucosamine were not down-regulated in HC treatment cells, suggesting that fructose-6-phosphate may be more likely converted to mannose-6-phosphate or glucose-6-phosphate, thereby leading to NDP-sugar production.

A second mechanism explaining the EPS response by *P. tricornutum* to high *p*CO_2_ levels could be that most genes encoding GTs and ABC transporter systems in the ER or Golgi bodies were up-regulated, which altered the polymerization steps of EPS synthesis (Figure 6, orange and purple frame). The Golgi apparatus is the major site of glycosylation reactions. In contrast, the NDP-sugar substrates of Golgi-associated glycosyltransferases are synthesized in the cytoplasm (Tiwari et al., 2016). Most genes encoding GTs were up-regulated, and increased expression of some ABC transporters indicate potentially increased activity in the Golgi apparatus leading to EPS secretion. These EPS then undergo further self-assembly in external environments and form cell frustule coatings, adhesive structures, or are used for motility (Wetherbee et al., 1998; Willis et al., 2015). The above two mechanistic responses were also observed as responses of *F. cylindrus* to extreme sea ice habitats (Aslam et al., 2018). However, the types of NDP-sugars involved were uniformly the same. For example, besides common NDP-sugars (e.g., GDP-Man, GDP-Fuc, UDP-GlcA, UDP-Glc, UDP-Gal, and UDP-GlcA), the gene encoding the enzymes involved in UDP-Xyl and UDP-Ara were also differentially expressed in response to HC condition. Thus, these combined results suggest that these metabolic pathways are likely common features of EPS production by diatom taxa in response to environmental signals, but some difference of local subdivision suggested these responses were closely related to algal species and environmental factors.

In addition to the above two mechanistic responses to environmental change, the results presented here improve our understanding of NDP-sugar transporters (NSTs) that are important for NDP-sugar interconversions (Figure 6, purple frame). An important function of NSTs is to transport NDP-sugars from the site of synthesis in the cytosol, across the membrane into the Golgi apparatus, and then supply substrates for GTs to synthesize polysaccharides and glycoproteins (Gerardy-Schahn et al., 2001; Zhang et al., 2011). NSTs were originally described in animal cells but are present throughout all eukaryotes that have been evaluated (Hirschberg et al., 1998). Changes in NST expression can affect glycoprotein biosynthesis and then indirectly affect polysaccharide levels on coat surfaces (Hadley et al., 2014). A total of eight genes encoding putative NSTs were identified in the *P. tricornutum* genome, of which seven were significantly up-regulated, implicating their role in EPS over-production in response to elevated *p*CO_2_ levels.

## Conclusion

In summary, our study showed that elevated *p*CO_2_ levels facilitate the increased release of EPS from *P. tricornutum* diatom cells and alter their chemical compositions. These data suggest that EPS release is likely due to carbon fixation outpacing carbon needed for growth, leading to the over-synthesis of carbon-rich storage products. NDP-sugar activation and accelerated glycosylation are two possible mechanisms that could explain the increased EPS production in response to environmental changes. Further, up-regulation of NDP-sugar transporters could also play an important role in EPS over-production in response to elevated *p*CO_2_ levels. This is the first report to show that both molecular and metabolic plasticity is required of *P. tricornutum* to cope with elevated *p*CO_2_ levels. These results provide a more resolved model for the production of EPS by diatoms and contribute to a better understanding of the response of EPS metabolism to environmental signals, and especially increased *p*CO_2_ levels.

## Data Availability Statement

The RNA-seq data were deposited in the ArrayExpress database (www.ebi.ac.uk/arrayexpress) under accession number E-MTAB-8351.

## Author Contributions

XXZ were responsible for the conception and design of this research. WZ, YY, and XZ were responsible for data collection and collation. WZ responsible for analysis, interpretation, and manuscript content. The approval of XT was obtained prior to article submission and provided financial support for this research.

## Conflict of Interest

The authors declare that the research was conducted in the absence of any commercial or financial relationships that could be construed as a potential conflict of interest.

## Footnotes

1 http://protists.ensembl.org/Phaeodactylum_tricornutum/Info/Index

2 www.cazy.org

3 www.ebi.ac.uk/arrayexpress

## References

[B1] Abdullahi A. S. Underwood G. J. C. Gretz M. R. (2006). Extracellular matrix assembly in diatoms (bacillariophyceae). v. environmental effects on polysaccharide synthesis in the model diatom, *Phaeodactylum tricornutum* 1. *J. Phycol.* 42 363–378. 10.1111/j.1529-8817.2006.00193.x

[B2] Ai X.-X. Liang J.-R. Gao Y.-H. Lo S. C.-L. Lee F. W.-F. Chen C.-P. (2015). MALDI-TOF MS analysis of the extracellular polysaccharides released by the diatom *Thalassiosira pseudonana* under various nutrient conditions. *J. Appl. Phycol.* 27 673–684. 10.1007/s10811-014-0360-0

[B3] Anders S. Huber W. (2012). *Differential Expression of RNA-Seq Data at the Gene Level–the DESeq Package.* Heidelberg: European Molecular Biology Laboratory (EMBL).

[B4] Anders S. Pyl P. T. Huber W. (2015). HTSeq—a Python framework to work with high-throughput sequencing data. *Bioinformatics* 31 166–169. 10.1093/bioinformatics/btu638 25260700 PMC4287950

[B5] Aslam S. N. Cresswell−Maynard T. Thomas D. N. Underwood G. J. C. (2012). Production and characterization of the intra−and extracellular carbohydrates and polymeric substances (EPS) of three sea−ice diatom species, and evidence for a cryoprotective role for EPS. *J. Phycol.* 48 1494–1509. 10.1111/jpy.12004 27009999

[B6] Aslam S. N. Strauss J. Thomas D. N. Mock T. Gjc U. (2018). Identifying metabolic pathways for production of extracellular polymeric substances by the diatom *Fragilariopsis cylindrus* inhabiting sea ice. *ISME J.* 12 1237–1251. 10.1038/s41396-017-0039-z 29348581 PMC5932028

[B7] Berman-Frank I. Dubinsky Z. (1999). Balanced growth in aquatic plants: myth or reality? Phytoplankton use the imbalance between carbon assimilation and biomass production to their strategic advantage. *Bioscience* 49 29–37. 10.1525/bisi.1999.49.1.29

[B8] Bolger A. M. Lohse M. Usadel B. (2014). Trimmomatic: a flexible trimmer for Illumina sequence data. *Bioinformatics* 30 2114–2120. 10.1093/bioinformatics/btu170 24695404 PMC4103590

[B9] Calazans G. M. T. Lima R. C. de França F. P. Lopes C. E. (2000). Molecular weight and antitumour activity of *Zymomonas mobilis* levans. *Int. J. Biol. Macromol.* 27 245–247. 10.1016/s0141-8130(00)00125-2 10921850

[B10] Czaczyk K. Myszka K. (2007). Biosynthesis of extracellular polymeric substances (EPS) and its role in microbial biofilm formation. *Pol. J. Environ. Stud.* 16 799–806.r

[B11] de Brouwer J. F. Wolfstein K. Ruddy G. K. Jones T. E. Stal L. J. (2005). Biogenic stabilization of intertidal sediments: the importance of extracellular polymeric substances produced by benthic diatoms. *Microb. Ecol.* 49 501–512. 10.1007/s00248-004-0020-z 16052376

[B12] de Brouwer J. F. C. Ruddy G. K. Jones T. E. R. Stal L. J. (2002). Sorption of EPS to sediment particles and the effect on the rheology of sediment slurries. *Biogeochemistry* 61 57–71. 10.1016/j.watres.2009.06.031 19595428

[B13] Decho A. W. Gutierrez T. (2017). Microbial extracellular polymeric substances (epss) in ocean systems. *Front. Microbiol.* 8:922. 10.3389/fmicb.2017.00922 28603518 PMC5445292

[B14] Decho A. W. Luoma S. N. (1994). Humic and fulvic acids: sink or source in the availability of metals to the marine bivalves *Macoma balthica* and *Potamocorbula amurensis*? *Mar. Ecol. Prog. Ser.* 108:133 10.3354/meps108133

[B15] Deming J. W. (2010). Sea ice bacteria and viruses. *Sea Ice* 2 247–282. 10.1002/9781444317145.ch7

[B16] Doney S. C. Fabry V. J. Feely R. A. Kleypas J. A. (2009). Ocean acidification: the other CO2 problem. *Annu. Rev. Mar. Sci.* 1 169–192. 10.1146/annurev.marine.010908.163834 21141034

[B17] Donot F. Fontana A. Baccou J. C. Schorr-Galindo S. (2012). Microbial exopolysaccharides: main examples of synthesis, excretion, genetics and extraction. *Carbohydr. Polym.* 87 951–962. 10.1016/j.carbpol.2011.08.083

[B18] Fu F. X. Tatters A. O. Hutchins D. A. (2012). Global change and the future of harmful algal blooms in the ocean. *Mar. Ecol. Prog. Ser.* 470 207–233. 10.3354/meps10047

[B19] Gao K. Campbell D. A. (2014). Photophysiological responses of marine diatoms to elevated CO2 and decreased pH: a review. *Funct. Plant. Biol.* 41 449–459. 10.1071/fp13247 32481004

[B20] Gao K. Helbling E. W. Häder D.-P. Hutchins D. A. (2012). Responses of marine primary producers to interactions between ocean acidification, solar radiation, and warming. *Mar. Ecol. Prog. Ser.* 470 167–189. 10.3354/meps10043

[B21] Gerardy-Schahn R. Oelmann S. Bakker H. (2001). Nucleotide sugar transporters: biological and functional aspects. *Biochimie* 83 775–782. 10.1016/s0300-9084(01)01322-0 11530210

[B22] Gu Z. Eils R. Schlesner M. (2016). Complex heatmaps reveal patterns and correlations in multidimensional genomic data. *Bioinformatics* 32 2847–2849. 10.1093/bioinformatics/btw313 27207943

[B23] Gügi B. le Costaouec T. Burel C. Lerouge P. Helbert W. Bardor M. (2015). Diatom-specific oligosaccharide and polysaccharide structures help to unravel biosynthetic capabilities in diatoms. *Mar. Drugs* 13 5993–6018. 10.3390/md13095993 26393622 PMC4584364

[B24] Guillard R. R. L. (1975). “Culture of phytoplankton for feeding marine invertebrates,” in *Culture of Marine Invertebrate Animals*, eds Smith W. L. Chanley M. H., (Boston, MA: Springer), 29–60. 10.1007/978-1-4615-8714-9_3

[B25] Hadley B. Maggioni A. Ashikov A. Day C. J. Haselhorst T. Tiralongo J. (2014). Structure and function of nucleotide sugar transporters: current progress. *Comput. Struct. Biotechnol. J.* 10 23–32. 10.1016/j.csbj.2014.05.003 25210595 PMC4151994

[B26] Hessen D. O. Anderson T. R. (2008). Excess carbon in aquatic organisms and ecosystems: physiological, ecological, and evolutionary implications. *Limnol. Oceanogr.* 53 1685–1696. 10.4319/lo.2008.53.4.1685

[B27] Hirschberg K. Miller C. M. Ellenberg J. Presley J. F. Siggia E. D. Phair R. D. (1998). Kinetic analysis of secretory protein traffic and characterization of Golgi to plasma membrane transport intermediates in living cells. *J. Cell Biol.* 143 1485–1503. 10.1083/jcb.143.6.1485 9852146 PMC2132993

[B28] Hong H. Shen R. Zhang F. Wen Z. Chang S. Lin W. (2017). The complex effects of ocean acidification on the prominent N2-fixing cyanobacterium *Trichodesmium*. *Science* 356 527–531. 10.1126/science.aao0428 28450383

[B29] Hutchins D. A. Fu F.-X. Zhang Y. Warner M. E. Feng Y. Portune K. (2007). CO2 control of *Trichodesmium* N2 fixation, photosynthesis, growth rates, and elemental ratios: implications for past, present, and future ocean biogeochemistry. *Limnol. Oceanogr.* 52 1293–1304. 10.4319/lo.2007.52.4.1293

[B30] Jin P. Wang T. Liu N. Dupont S. Beardall J. Boyd P. W. (2015). Ocean acidification increases the accumulation of toxic phenolic compounds across trophic levels. *Nat. Commun.* 6:8714. 10.1038/ncomms9714 26503801 PMC4640080

[B31] Kaplan D. (2013). Absorption and adsorption of heavy metals by microalgae. Handbook of microalgal culture. *Appl. Phycol. Biotechnol.* 2 602–611. 10.1002/9780470995280.ch26

[B32] Kim D. Langmead B. Salzberg S. L. (2015). HISAT: a fast spliced aligner with low memory requirements. *Nat. Methods* 12 357–360. 10.1038/nmeth.3317 25751142 PMC4655817

[B33] Kooistra W. H. De Stefano M. Mann D. G. Medlin K. (2003). “The Phylogeny of the Diatoms,” in *Silicon Biomineralization. Progress in Molecular and Subcellular Biology*, Vol. 33 ed. Müller W. E. G. (Berlin: Springer). 10.1007/978-3-642-55486-5_3 14518369

[B34] Kroth P. G. Chiovitti A. Gruber A. Martinjezequel V. Mock T. Parker M. S. (2008). A model for carbohydrate metabolism in the diatom *Phaeodactylum tricornutum* deduced from comparative whole genome analysis. *PLoS One* 3:e1426. 10.1371/journal.pone.0001426 18183306 PMC2173943

[B35] Macías-Sánchez M. D. Mantell C. Rodríguez M. Ossa E. M. D. la, Lubián L. M. Montero O. (2007). Supercritical fluid extraction of carotenoids and chlorophyll a from *Synechococcus* sp. *J. Supercrit. Fluids* 39 323–329. 10.1016/j.supflu.2006.03.008 16332118

[B36] Magaletti E. Urbani R. Sist P. Ferrari C. R. Cicero A. M. (2004). Abundance and chemical characterization of extracellular carbohydrates released by the marine diatom *Cylindrotheca fusiformis* under N-and P-limitation. *European J. Phycol.* 39 133–142. 10.1080/0967026042000202118

[B37] Mcconville M. J. Mitchell C. Wetherbee R. (1985). Patterns of carbon assimilation in a microalgal community from annual sea ice, east Antarctica. *Polar. Biol.* 4 135–141. 10.1007/bf00263876

[B38] Middelburg J. J. Barranguet C. Boschker H. T. S. Herman P. M. J. Moens T. Heip C. H. R. (2000). The fate of intertidal microphytobenthos carbon: an in situ 13C−labeling study. *Limnol. Oceanogr.* 45 1224–1234. 10.4319/lo.2000.45.6.1224

[B39] Millero F. J. Huang F. Williams N. Waters J. Woosley R. (2009). The effect of composition on the density of South Pacific Ocean waters. *Mar. Chem.* 114 56–62. 10.1016/j.marchem.2009.04.001

[B40] Mishra A. Jha B. (2009). Isolation and characterization of extracellular polymeric substances from micro-algae *Dunaliella salina* under salt stress. *Bioresour. Technol.* 100 3382–3386. 10.1016/j.biortech.2009.02.006 19272770

[B41] Mitchell A. L. Attwood T. K. Babbitt P. C. Blum M. Bork P. Bridge A. (2018). InterPro in 2019: improving coverage, classification and access to protein sequence annotations. *Nucleic Acids Res.* 47 D351–D360. 10.1093/nar/gky1100 30398656 PMC6323941

[B42] Nielsen H. (2017). Predicting secretory proteins with SignalP. *Methods Mol. Biol.* 1611 59–73. 10.1007/978-1-4939-7015-5_6 28451972

[B43] Oksanen J. Blanchet F. G. Kindt R. Legendre P. O’hara R. B. Simpson G. L. (2003). VEGAN, a package of R functions for community ecology. *J. Veg. Sci.* 14 927–930. 10.1111/j.1654-1103.2003.tb02228.x

[B44] Porra R. J. Thompson W. A. Kriedemann P. E. (1989). Determination of accurate extinction coefficients and simultaneous equations for assaying chlorophylls a and b extracted with four different solvents: verification of the concentration of chlorophyll standards by atomic absorption spectroscopy. *Biochim. Biophys. Acta Bioenerg.* 975 384–394. 10.1016/S0005-2728(89)80347-0

[B45] Riebesell U. Gattuso J.-P. (2014). Lessons learned from ocean acidification research. *Nat. Clim. Chang.* 5 12–14. 10.1038/nclimate2456 28877955

[B46] Scala S. Carels N. Falciatore A. Chiusano M. L. Bowler C. (2002). Genome properties of the diatom *Phaeodactylum tricornutum*. *Plant Physiol.* 129 993–1002. 10.1104/pp.010713 12114555 PMC166495

[B47] Shi D. Hong H. Su X. Liao L. Chang S. Lin W. (2019). The physiological response of marine diatoms to ocean acidification: differential roles of seawater *p*CO2 and pH. *J. Phycol.* 55 521–533. 10.1111/jpy.12855 30849184

[B48] Siaut M. Heijde M. Mangogna M. Montsant A. Coesel S. Allen A. (2007). Molecular toolbox for studying diatom biology in *Phaeodactylum tricornutum*. *Gene* 406 23–35. 10.1016/j.gene.2007.05.022 17658702

[B49] Simon M. Grossart H.-P. Schweitzer B. Ploug H. (2002). Microbial ecology of organic aggregates in aquatic ecosystems. *Aquat. Microb. Ecol.* 28 175–211. 10.3354/ame028175

[B50] Song W. Zhao C. Mu S. Pan X. Zhang D. Al-Misned F. A. (2015). Effects of irradiation and pH on fluorescence properties and flocculation of extracellular polymeric substances from the cyanobacterium *Chroococcus minutus*. *Colloids Surf. B* 128 115–118. 10.1016/j.colsurfb.2015.02.017 25731101

[B51] Sonnenschein E. C. Gärdes A. Seebah S. Torres-Monroy I. Grossart H.-P. Ullrich M. S. (2011). Development of a genetic system for *Marinobacter adhaerens* HP15 involved in marine aggregate formation by interacting with diatom cells. *J. Microbiol. Methods* 87 176–183. 10.1016/j.mimet.2011.08.008 21880271

[B52] Steele D. J. Franklin D. J. Underwood G. J. C. (2014). Protection of cells from salinity stress by extracellular polymeric substances in diatom biofilms. *Biofouling* 30 987–998. 10.1080/08927014.2014.960859 25268215 PMC4706044

[B53] Sutherland I. W. (1999). “Biofilm Exopolysaccharides,” in *Microbial Extracellular Polymeric Substances*, eds Wingender J. Neu T. R. Flemming H. C. (Berlin: Springer). 10.1007/978-3-642-60147-7_4

[B54] Taylor J. D. McKew B. A. Kuhl A. McGenity T. J. Underwood G. J. C. (2013). Microphytobenthic extracellular polymeric substances (EPS) in intertidal sediments fuel both generalist and specialist EPS−degrading bacteria. *Limnol. Oceanogr.* 58 1463–1480.

[B55] Tiwari P. Sangwan R. S. Sangwan N. S. (2016). Plant secondary metabolism linked glycosyltransferases: an update on expanding knowledge and scopes. *Biotechnol. Adv.* 34 714–739. 10.1016/j.biotechadv.2016.03.006 27131396

[B56] Trapnell C. Williams B. A. Pertea G. Mortazavi A. Kwan G. van Baren M. J. (2010). Transcript assembly and quantification by RNA-Seq reveals unannotated transcripts and isoform switching during cell differentiation. *Nat. Biotechnol.* 28 511–515. 10.1038/nbt.1621 20436464 PMC3146043

[B57] Underwood G. J. C. Aslam S. N. Michel C. Niemi A. Norman L. Meiners K. M. (2013). Broad-scale predictability of carbohydrates and exopolymers in Antarctic and Arctic sea ice. *Proc. Natl. Acad. Sci. U.S.A.* 110 15734–15739. 10.1073/pnas.1302870110 24019487 PMC3785782

[B58] Underwood G. J. C. Boulcott M. Raines C. A. Waldron K. (2004). Environmental effects on exopolymer production by marine benthic diatoms: dynamics, changes in composition, and pathways of production1. *J. Phycol.* 40 293–304. 10.1111/j.1529-8817.2004.03076.x

[B59] Underwood G. J. C. Paterson D. M. (2003). The importance of extracellular carbohydrate productionby marine epipelic diatoms. *Adv. Bot. Res.* 40 183–240. 10.1016/S0065-2296(05)40005-1

[B60] Wetherbee R. Lind J. L. Burke J. Quatrano R. S. (1998). Minireview—the first kiss: establishment and control of initial adhesion by raphid diatoms. *J. Phycol.* 34 9–15. 10.1046/j.1529-8817.1998.340009.x

[B61] Wickham H. (2016). *ggplot2: Elegant Graphics for Data Analysis.* Berlin: Springer.

[B62] Willis A. Easonhubbard M. Hodson O. Maheswari U. Bowler C. Wetherbee R. (2015). Adhesion molecules from the diatom *Phaeodactylum tricornutum* (Bacillariophyceae): genomic identification by amino-acid profiling and in vivo analysis. *J. Phycol.* 50 837–849. 10.1111/jpy.12214 26988639

[B63] Wu Y. Gao K. Riebesell U. (2010). CO2-induced seawater acidification affects physiological performance of the marine diatom *Phaeodactylum tricornutum*. *Biogeosciences* 7 2915–2923. 10.5194/bg-7-2915-2010

[B64] Zhang B. Liu X. Qian Q. Liu L. Dong G. Xiong G. (2011). Golgi nucleotide sugar transporter modulates cell wall biosynthesis and plant growth in rice. *Proc. Natl. Acad. Sci. U.S.A.* 108 5110–5115. 10.1073/pnas.1016144108 21383162 PMC3064376

