# Supplementary material for: Elevated pCO2 Level Affects the Extracellular Polymer Metabolism of Phaeodactylum tricornutum
Source: Front Microbiol. 2020 Mar 4;11:339. doi: 10.3389/fmicb.2020.00339 (PMC7064563; doi:10.3389/fmicb.2020.00339)

**Supplementary Material**

Supplementary Table S1. Seawater dissolved inorganic carbon characteristics in the LC and HC treatments, before and after partial renewal of the medium in semi-continuous cultures. Total inorganic carbon (DIC), pH, salinity, nutrient concentrations, and temperature were used to drive all other parameters using a CO_2_ system and analyzing software (CO_2_SYS).

|  | Group | pH_NBS_ | DIC  (μmol kg^-1^) | | HCO_3_^-^  (μmol kg^-1^) | CO_3_^2-^  (μmol kg^-1^) | CO_2_  (μmol kg^-1^) | TA  (μmol kg^-1^) |
| --- | --- | --- | --- | --- | --- | --- | --- | --- |
| Before renewal | LC | 8.16 ± 0.04 | 2,772.78 ± 28.13 | 2,526.71 ± 25.25 | | 227.35 ± 2.82 | 18.71 ± 0.17 | 3,054.84 ± 30.87 |
|  | HC | 7.82 ± 0.03 | 2,989.44 ± 13.35 | 2,827.77 ± 13.20 | | 115.83 ± 1.32 | 45.84 ± 0.83 | 3,096.46 ± 12.15 |
| After renewal | LC | 8.16 ± 0.04 | 2,749.44 ± 10.28 | 2,507.32 ± 9.52 | | 223.38 ± 0.90 | 18.73 ± 0.11 | 3,026.95 ± 10.82 |
|  | HC | 7.83 ± 0.03 | 2,954.44 ± 6.46 | 2,975.66 ± 4.88 | | 114.77 ± 2.48 | 45.01 ± 0.76 | 3,061.39 ± 10.10 |

DIC, total dissolved inorganic carbon; TA, total alkalinity. Data are shown as the means ± SD (n=3).

Supplementary Table S2. Sequencing data quality and sequence read alignment analysis.

| Sample | Multiple mapped | Uniquely mapped | Non-splice reads | Splice reads | Raw_bases | Clean_bases | Q30 | GC |
| --- | --- | --- | --- | --- | --- | --- | --- | --- |
| HC_1 | 1,227,206(2.62%) | 42,313,736(90.39%) | 38,979,693(83.26%) | 3,334,043(7.12%) | 7.34G | 6.87G | 93.59% | 51.96% |
| HC_2 | 1,110,104(2.89%) | 34,591,674(90.11%) | 31,888,065(83.07%) | 2,703,609(7.04%) | 6.03G | 5.63G | 93.43% | 51.90% |
| HC_3 | 1,241,537(2.64%) | 42,590,825(90.41%) | 39,191,913(83.19%) | 3,398,912(7.21%) | 7.38G | 6.91G | 93.67% | 51.86% |
| LC_1 | 683,318(1.65%) | 35,403,804(85.70%) | 33,541,306(81.19%) | 1,862,498(4.51%) | 6.47G | 6.07G | 93.73% | 51.58% |
| LC_2 | 747,956(1.66%) | 38,388,594(85.14%) | 36,370,707(80.66%) | 2,017,887(4.48%) | 7.07G | 6.62G | 93.61% | 51.48% |
| LC_3 | 733,882(1.66%) | 37,900,924(85.67%) | 35,907,839(81.17%) | 1,993,085(4.51%) | 6.94G | 6.49G | 93.59% | 51.60% |

Raw_reads, original reads; Raw_bases, number of bases in the original sequencing data; Clean_reads, the number of clean reads obtained after filtering; Clean_bases, the number of bases in clean reads after filtering; valid_base, valid base percentage; Q30, the percentage of bases with a Qphred value > 30 as a percentage of the total number of bases; GC, total G + C content in the clean bases as a percentage of the total number of bases. For read mapping analyses: Total reads, the number of reads after sequence filtering (e.g., the clean reads); Total mapped, the number of sequences mapped to the genome; Multiple mapped, the number of sequences with multiple alignment positions on the reference genome; Uniquely mapped, the number of sequences with unique alignment positions on the reference genome; Splice reads: uniquely mapped reads that were segmentally aligned two exons (also known as Junction reads) where non-splice reads show the sequences aligned to the exon with reads mapped in proper pairs and the number of sequences on the double-ended alignment.

Supplementary Table S3. RT-qPCR primers and amplification results.

| Gene ID | Forward Primer | Reverse Primer | Annotation | Foldchange (Sequencing) | Foldchange (RT-qPCR) |
| --- | --- | --- | --- | --- | --- |
| Phatr3_J55126 | ACAAAGGAATGGTTACGGGAG | GGTAGTCGGAAAGGGTAGCA | PFP pyrophosphate dependent phosphofructokinase | 2.9152354 | 2.419626 |
| Phatr3_J14792 | CGGATGCTGGTATTGACGATG | ACCGTTCTTGGTGAGGGATT | Phosphoglycerate kinase | 9.1352449 | 4.568972 |
| Phatr3_J14284 | CAACCCGTCATTCTCACTCC | GCGTTCCGTCTCCTCCAAT | Pyrophosphate-dependent phosphofructose kinase | 1.9624363 | 1.038999 |
| Phatr3_EG02613 | ACGCCAATACTAACAGCCTAA | GTTTCCGCCCGCATCTTG | UDP-Glucose-Pyrophosphorylase/Phosphoglucomutase | 3.4211801 | 1.386074 |
| Phatr3_J46117 | CGACGCCAAGGTAGCAGTTC | AAACGAAGAAAGCCCACGAC | Alpha-(1,6)-fucosyltransferase | 1.6957563 | 1.31483 |
| Phatr3_J10693 | AACGCTGACATTGGACGCT | ATGGTGACTTTTGGTTTCTTGG | Mannose-6-phosphate isomerase | 4.1064857 | 2.842845 |
| Phatr3_J51128 | GGGACTTACGCCGAGGAT | AAGGAGCCGAGATGACAACC | Glyceraldehyde-3-phosphate dehydrogenase | 20.703439 | 13.84413 |
| Phatr3_EG02209 | TGTTTGCCGACGACTGCTC | CCGCTTGGTATGATGGGTATT | Phosphofructokinase | 1.5702402 | 1.28709 |
| [Phatr3_J29456](http://protists.ensembl.org/Phaeodactylum_tricornutum/Gene/Summary?g=Phatr3_J29456;r=16:730132-731560;t=Phatr3_J29456.t1;db=core) | TCAGAAGGACCGATGAAGGG | AGCAGAGGCGTCCACAATAG | Glyceraldehyde-3-phosphate dehydrogenase | 18.088899 | 8.886687 |
| Phatr3_J23924 | ACCTGGAAAGGGCATACGG | GCGTGGTGAAGGTTTTACTGG | Glucose-6-phosphate isomerase | 1.6537784 | 1.007732 |
| Phatr3_J5629 | TGGAGAGAGATTGTGGCGGA | GGAATGGCGGTGGGTATGT | Phosphoglycerate mutase | 1.6248949 | 1.266071 |
| Phatr3_EG02409 | CCGACAACGAGAACCCCATT | AGTAGAGATACCACCCGCCT | Phosphoglycerate mutase | 1.5894873 | 0.934983 |
| Phatr3_J14994 | AGGAAAAACCGACGAGAACG | CATAATACAGAGTCCCCACGAAC | Glutamine-fructose-6-phosphate transaminase | 1.7958399 | 1.266071 |
| Phatr3_J25417 | CGGGCTTGGAACAGTCAG | CAACAGCATAAGGGGAACGG | Gdp-mannose 4,6-dehydratase | 2.9717566 | 1.795112 |
| Phatr3_J15495 | CGGGAGATGAACAAGGCAAG | CCATAACAGCAAACAGAGGGATAC | Glucokinase | 1.8155452 | 1.694853 |

Supplementary Table S4. Gene identifiers, abbreviations, and annotations for 115 EPS metabolism-related genes.

| Gene | Gene ID | Enzyme name |
| --- | --- | --- |
| Phatr3_EG00041 | UXS1 | NAD-dependent epimerase/dehydratase |
| Phatr3_EG00115 | B3GALT5 | Beta-1,3-galactosyltransferase 5 |
| Phatr3_EG00407 | GMH3 | Alpha-1,2-galactosyltransferase |
| Phatr3_EG01004 | MNDA | Beta-mannosidase |
| Phatr3_EG01211 | RHM2 | UDP-4-keto-6-deoxy-glucose-3,5-epimerase |
| Phatr3_EG01605 | GMS1 | UDP-galactose transmembrane transport |
| Phatr3_EG01613 | MPI | Mannose-6-phosphate isomerase |
| Phatr3_EG01961 | ABCA1 | ATP-binding cassette sub-family A member 1 |
| Phatr3_EG02208 | G6PI | Glucose-6-phosphate isomerase |
| Phatr3_EG02351 | PSL5 | Alpha subunit of glucosidase |
| Phatr3_EG02374 | MNS2 | Alpha-1,2-Mannosidase |
| Phatr3_EG02430 | LAC4 | Beta-galactosidase |
| Phatr3_EG02432 | LAC4 | Beta-galactosidase |
| Phatr3_EG02613 | G6PI | UDP-Glucose-Pyrophosphorylase/Phosphoglucomutase |
| Phatr3_J10693 | MPI | Mannose-6-phosphate isomerase |
| Phatr3_J10976 | ALG3 | Alpha-1,3-mannosyltransferase |
| Phatr3_J11559 | GAE5 | NAD-dependent epimerase/dehydratase |
| Phatr3_J11674 | ABCB25 | ABC transporter B family member 25 |
| Phatr3_J12401 | GATase4 | Glutamine amidotransferase |
| Phatr3_J12984 | GAE1 | UDP-glucuronate 4-epimerase |
| Phatr3_J13741 | PIGU | Phosphatidylinositol glycan anchor biosynthesis class U protein |
| Phatr3_J14002 | TUN | UDP-glycosyltransferase TURAN |
| Phatr3_J14444 | ALG14 | UDP-n-acetylglucosamine n-acetylglucosaminyltransferase |
| Phatr3_J14994 | GLMS | Glutamine-fructose-6-phosphate transaminase |
| Phatr3_J15125 | FCL | GDP-4-keto-6-deoxy-D-mannose-3,5-epimerase-4-reductase |
| Phatr3_J15495 | GLK | Glucokinase |
| Phatr3_J16195 | RLF | Cytochrome b5 domain-containing protein RLF |
| Phatr3_J16490 | NIA | Genome polyprotein |
| Phatr3_J16571 | PGM1 | Phosphoglucomutase |
| Phatr3_J16722 | G6PI | Glucose-6-phosphate isomerase |
| Phatr3_J18303 | GPI14 | Mannosyl transferase |
| Phatr3_J18745 | UGDH | UDP-glucose 6-dehydrogenase |
| Phatr3_J19705 | DPM1 | Dolichyl-phosphate mannosyltransferase |
| Phatr3_J20026 | ABCB25 | ABC transporter B family member 25 |
| Phatr3_J20120 | GMS1 | UDP-galactose transmembrane transport |
| Phatr3_J21201 | SQD1 | UDP-sulfoquinovose synthase, plastid |
| Phatr3_J21548 | ABCB9 | ATP-binding cassette sub-family B member 9 |
| Phatr3_J22554 | ALG2 | Mannosyltransferase |
| Phatr3_J22739 | GMS1 | UDP-galactose transmembrane transport |
| Phatr3_J22921 | GMS1 | UDP-galactose transmembrane transport |
| Phatr3_J23497 | ABCA1 | ATP-binding cassette sub-family A member 1 |
| Phatr3_J23639 | USP | Precursor of phosphorylase UDP-glucose diphosphorylase |
| Phatr3_J23924 | G6PI | Glucose-6-phosphate isomerase |
| Phatr3_J25417 | GMD1 | GDP-mannose 4,6-dehydratase |
| Phatr3_J27190 | GALE | UDP-glucose 4-epimerase |
| Phatr3_J28882 | PMM | Phosphomannomutase |
| Phatr3_J29042 | NST | Nucleotide-sugar transporter |
| Phatr3_J29658 | MCR1 | NADH-cytochrome b5 reductase 2 |
| Phatr3_J29660 | MCR1 | NADH-cytochrome b5 reductase 3 |
| Phatr3_J30638 | GPI8 | GPI-anchor transamidase |
| Phatr3_J32708 | PGM1 | Mutase phosphoglucomutase |
| Phatr3_J3369 | GALAK | Galactokinase |
| Phatr3_J34317 | ALG5 | Dolichol phosphate glucosyltransferase |
| Phatr3_J34610 | GPI12 | N-acetylglucosaminyl phosphatidylinositol deacetylase |
| Phatr3_J34716 | GAE4 | NAD-dependent epimerase/dehydratase |
| Phatr3_J37908 | CHS2 | Transmembrane chitin synthase |
| Phatr3_J38642 | ABCB25 | ABC transporter B family member 25 |
| Phatr3_J39282 | ALFA | Alpha-L-fucosidase |
| Phatr3_J39480 | GLYT | Alpha-1,3-mannosyl-glycoprotein 2-beta-N-acetylglucosaminyltransferase |
| Phatr3_J39708 | ABCA1 | ATP-binding cassette sub-family A member 1 |
| Phatr3_J4234 | NST | Nucleotide-sugar transporter |
| Phatr3_J43101 | GPI2 | Phosphatidylinositol N-acetylglucosaminyltransferase subunit |
| Phatr3_J43491 | PGM1 | Phosphoglucomutase 1 |
| Phatr3_J44040 | SEC | UDP-N-acetylglucosamine--peptide N-acetylglucosaminyltransferase |
| Phatr3_J44117 | ALG6 | Dolichyl pyrophosphate Glc1Man9GlcNAc2 alpha-1,3-glucosyltransferase |
| Phatr3_J44338 | MNN10 | Alpha-1,6-mannosyltransferase |
| Phatr3_J44339 | GT7 | Glycosyltransferase 7 |
| Phatr3_J44406 | GALAK | Galacturonokinase |
| Phatr3_J44425 | ALG12 | Alpha-mannosyltransferase |
| Phatr3_J44574 | ALG9 | Dol-P-Man:Man(6)GlcNAc(2)-PP-Dol alpha-1,2-mannosyltransferase |
| Phatr3_J44759 | CHS2 | Transmembrane chitin synthase |
| Phatr3_J44790 | GPI10 | GPI mannosyltransferase 3 |
| Phatr3_J44905 | ALG6 | Dolichyl pyrophosphate Glc1Man9GlcNAc2 alpha-1,3-glucosyltransferase |
| Phatr3_J45039 | GLYT | Alpha-1,3-mannosyl-glycoprotein 2-beta-N-acetylglucosaminyltransferase |
| Phatr3_J45073 | HEXO1 | Beta-hexosaminidase 1 |
| Phatr3_J45434 | GME | NAD-dependent epimerase/dehydratase |
| Phatr3_J45496 | XYLT | Betaxylosyltransferase |
| Phatr3_J45980 | ALG5 | Dolichol phosphate glucosyltransferase |
| Phatr3_J46109 | FUT12 | Fucosyltransferase-like protein |
| Phatr3_J46110 | FUT11 | Core alphafucosyltransferase |
| Phatr3_J46117 | FUT | Fucosyltransferase-like protein |
| Phatr3_J46194 | GMS1 | UDP-galactose transmembrane transport |
| Phatr3_J46727 | GAE | UDP-glucuronate 4-epimerase |
| Phatr3_J46785 | GALE | UDP-glucose 4-epimerase |
| Phatr3_J47152 | RHM2 | UDP-glucose 4,6-dehydratase/UDP-4-keto-6-deoxy-D-glucose 3,5-epimerase/UDP-4-keto-L-rhamnose-reductase |
| Phatr3_J47282 | GAA1 | Glycosylphosphatidylinositol anchor attachment 1 |
| Phatr3_J47349 | FUT | Fucosyltransferase-like protein |
| Phatr3_J47390 | GPI13 | GPI ethanolamine phosphate transferase 3 |
| Phatr3_J48173 | RRP45A | Exosome complex component |
| Phatr3_J48865 | ALFA | Alpha-L-fucosidase |
| Phatr3_J49563 | HEXO3 | Beta-hexosaminidase 3 |
| Phatr3_J49631 | MNT3 | Alpha-1,3-mannosyltransferase |
| Phatr3_J49836 | GPI16 | GPI transamidase component |
| Phatr3_J49849 | NST | Nucleotide-sugar transporter |
| Phatr3_J52108 | GMII | Alpha mannosidase |
| Phatr3_J52248 | GMII | Alpha mannosidase |
| Phatr3_J52551 | GPI1 | Phosphatidylinositol N-acetylglucosaminyltransferase subunit Q |
| Phatr3_J52603 | PAGM | Phosphoacetylglucosamine mutase |
| Phatr3_J54068 | PIGA | N-acetylglucosaminyl-phosphatidylinositol biosynthetic protein |
| Phatr3_J54493 | USP1 | UDP-n-acetylglucosamine diphosphorylase |
| Phatr3_J54536 | NEG1 | Endo-1,6-beta-D-glucanase |
| Phatr3_J54588 | GWT1 | GPI-anchored wall transfer protein 1 |
| Phatr3_J54621 | ALG11 | Alpha-1,2-mannosyltransferase ALG11 |
| Phatr3_J54703 | QRI1 | UDP-n-acetylglucosamine pyrophosphorylase |
| Phatr3_J54844 | GNTI | Alpha-1,3-mannosyl-glycoprotein 2-beta-N-acetylglucosaminyltransferase |
| Phatr3_J55197 | STT3 | Dolichyl-diphosphooligosaccharide--protein glycosyltransferase subunit STT3 |
| Phatr3_J55198 | STT3 | Dolichyl-diphosphooligosaccharide--protein glycosyltransferase subunit STT4 |
| Phatr3_J5857 | G6PI | Glucose-6-phosphate isomerase |
| Phatr3_J6435 | RLF | Cytochrome b5 domain-containing protein RLF |
| Phatr3_J7426 | RBG4 | Glycine-rich RNA-binding protein 4 |
| Phatr3_J9427 | ALG13 | UDP-n-acetylglucosamine transferase subunit alg13 |
| Phatr3_J9724 | GPT2 | UDP-N-acetylglucosamine--dolichyl-phosphate N-acetylglucosaminephosphotransferase |
| Phatr3_Jdraft1431 | FUK | L-fucose kinase |
| Phatr3_Jdraft1613 | ABCB11 | ATP-binding cassette sub-family B member 11 |
| Phatr3_Jdraft1777 | CHS | Chitin synthase |

Supplementary Figure 1. Hierarchical clustering of expression values (mean fragments per kilobase of transcript per million mapped reads; FPKM) among samples for 269 genes encoding carbohydrate-active enzymes in the *Phaeodactylum tricornutum* genome (<http://protists.ensembl.org/Phaeodactylum_tricornutum/Info/Index)>. The enzymes were identified based on the CArbohydrate-Active enZYmes (CAZy) database. The color scale ranges from saturated red indicating highly expressed genes to saturated blue indicating weakly expressed genes, while white indicates relatively moderate expression. The heatmap was generated using the ComplexHeatmap program (Gu et al., 2016) using k-means clustering with a one minus Pearson correlation distance metric and average linkage method to cluster genes into a dendrogram.


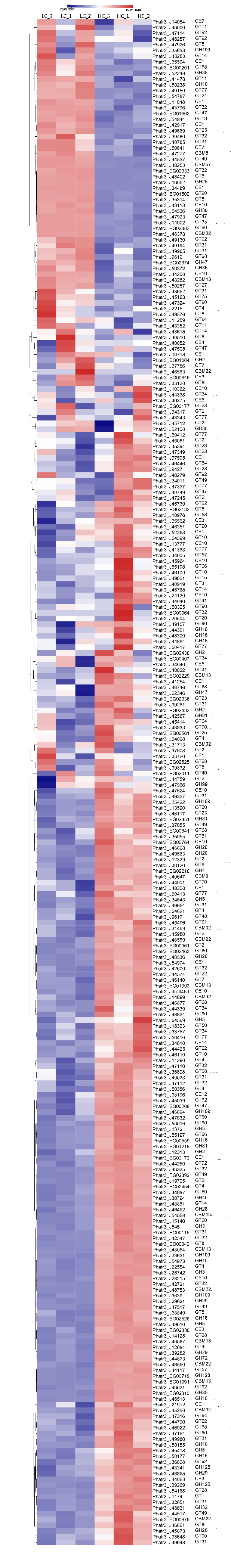


Supplementary Figure 2:

Figure R1: Detection of RNA integrity in *P.tricornutum* samples.(A) Gel electrophoresis result; (10) Peak map from Aglient 2100.

R1808249, R1808250, R1808251, R1808252, R1808253 and R1808254 are sample LC_1, LC_2, LC_3, HC_1, HC_2, HC_3, respectively


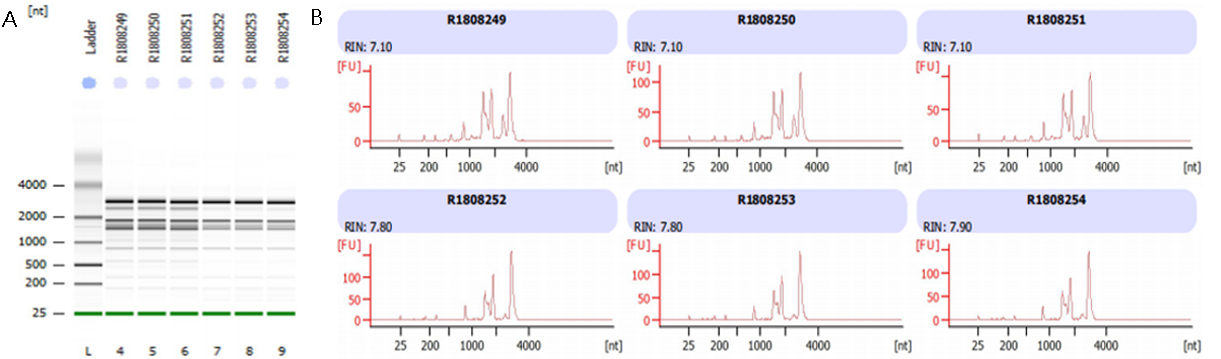

Supplement: Supplementary file 1 [file Data_Sheet_1.docx]
